# Functional analyses of *Apis cerana cerana DnaJC28*: a new biomarker for environmental stress

**DOI:** 10.3389/fphys.2026.1945469

**Published:** 2026-10-23

**Authors:** Guilin Li, Xinyi Zou, Shaojuan Yin, Yue Sun, Qianqian Wen

**Affiliations:** 1 College of Life Sciences, Qufu Normal University, Qufu, China; 2 Shandong Key Laboratory of Wetland Ecology and Biodiversity Conservation in the Lower Yellow River, Qufu, Shandong, China

**Keywords:** antioxidant, DnaJC28, honeybees, mechanism, stresses

## Abstract

Pollination by honeybees is vital for maintaining the health of both ecological and agricultural systems. However, honeybee health is often influenced by multiple environmental stresses. Therefore, identifying the key genes involved in stress responses and investigating the molecular mechanisms through which honeybees respond to environmental stress are crucial. In this study, we report that *Apis cerana cerana* DnaJC28 (AccDnaJC28) is a typical DnaJ protein belonging to the heat shock protein subfamily that contains a J domain and is localized in the cytoplasm. The mRNA levels of *AccDnaJC28* were higher in the thorax than in other tissues and in 1-day-old workers than in bees at other developmental stages. Several environmental stressors induced the expression of *AccDnaJC28*. Functional analyses revealed that silencing *AccDnaJC28* decreased the thermotolerance of *A. cerana cerana*, whereas its overexpression increased the survival of *Drosophila* S2 cells under heat stress. These results indicate the crucial role of *AccDnaJC28* in the stress response and its potential suitability as a novel biomarker. Mechanistic analysis further revealed that knockdown of *AccDnaJC28* reduced both the enzymatic activity of certain antioxidant enzymes and the expression levels of related antioxidant genes. In addition, the upregulated expression of certain antioxidant genes may partially compensate for the functional deficiency caused by *AccDnaJC28* knockdown. Taken together, our findings demonstrate that *AccDnaJC28* is essential for the response of *A. cerana cerana* to environmental stress, as it mitigates oxidative damage via the antioxidant signaling pathway. These results contribute to the understanding of the foundational defense mechanisms initiated by honeybees in response to environmental stress.

## Introduction

Honeybees are important pollinators of many crops and wild plants worldwide and produce valuable products for humans (Klein et al., 2007; Bava et al., 2023; Farias et al., 2023; Requier et al., 2023). However, honeybee health and colony resilience are currently being negatively affected by multiple environmental stressors (Potts et al., 2010; Evans and Schwarz, 2011; Goulson et al., 2015). For example, cold stress at the capped brood stage impairs honeybee development, leading to increased misorientation and pupal and adult mortality (Wang et al., 2016). Exposure to heat stress during development influences the immunocompetence of drones, queens and workers (Medina et al., 2020), triggers fluctuations in forewing shape asymmetry, and decreases longevity and the age at onset of foraging in *Apis mellifera* L (Medina et al., 2018). Very low concentrations of paraquat interfere with the development of oenocytes in honeybee larvae (Cousin et al., 2013) and trigger honeybee death (Phokasem et al., 2024). Beta-cypermethrin has chronic toxic effects on honeybee larvae and compromises their fitness even at low concentrations (He et al., 2022). Abamectin negatively affects midgut cells, potentially leading to digestive dysfunction (Aljedani, 2017). Therefore, exploration of the molecular mechanisms through which honeybees resist environmental stress and the identification of key molecules, genes or proteins that could promote honeybee colony resilience in the presence of different environmental stressors are urgently needed.

Heat shock proteins (HSPs) are known to fold newly synthesized proteins, repair denatured proteins, and dispose of misfolded proteins (Becker and Craig, 1994; Feder and Hofmann, 1999). In addition to these functions, HSPs significantly protect organisms from environmental stressors and are well-established mediators and biomarkers of stress response (Feder and Hofmann, 1999; King and MacRae, 2015; Chen et al., 2018; Rajamanickam et al., 2025). HSPs can be divided into subtypes according to their molecular weight: sHSPs, HSP40, HSP60, HSP70, HSP90, and HSP100 (Srensen et al., 2010). Many species have the greatest number of genes encoding HSP40, which is often referred to as DnaJ because of the presence of a J domain (Dekker et al., 2015; Craig and Marszalek, 2017). On the basis of the presence or absence of a J domain, zinc-binding domain, glycine/phenylalanine-rich region, and C-terminal domain, DnaJ proteins are classified into the following distinct groups: DnaJA, DnaJB, and DnaJC proteins (Dekker et al., 2015). Notably, in some species, the number of *DnaJC* genes is significantly greater than that of *DnaJA* and *DnaJB* genes (Kampinga et al., 2009; Li et al., 2020a).

Multiple studies have revealed the critical functions of DnaJs in different organisms. For example, DnaJC1 is a prognostic predictor of hepatocellular carcinoma. Its knockdown suppresses hepatocellular carcinoma cell proliferation, migration, and invasion while promoting apoptosis (Fan et al., 2023). Research has indicated that DnaJA2 is involved in controlling cell division and maintaining chromosomal integrity (Huang et al., 2023). Biallelic *DnaJC7* variants cause familial amyotrophic lateral sclerosis (Yamashita et al., 2025). The mitochondrial chaperonin DnaJC15 renders chemoresistant ovarian cancer cells more vulnerable to ferroptosis (Miglietta et al., 2025). Under heat stress conditions, a significant upregulation of *DnaJA4*, *DnaJB1*, and *DnaJB4* expression is observed in T cells (Chand et al., 2021). These findings indicate the functional diversity of DnaJ proteins.

The function of *DnaJC28*, a member of the DnaJ protein family, has been characterized in several species. Molecular evolution studies have indicated that mutations in *DnaJC28* may endow Chinese rhesus macaques with rapid thermoregulation capacity (Wang et al., 2025). DnaJC28 has also been suggested to be associated with sporadic late-onset Parkinson’s disease (Zhang et al., 2022). DnaJC28 affects feeding efficiency and is under positive selection in Angus cattle (Taye et al., 2018), whereas *DnaJC28* is a key candidate gene involved in tropical adaptation in Nellore cattle (Fernandes Junior et al., 2020). These results indicate that *DnaJC28* plays crucial roles in animals. However, related studies have focused mainly on a small number of mammals, and the function of *DnaJC28* in insects remains unknown.

*Apis cerana cerana* is a bee species unique to China that plays a crucial role in maintaining biodiversity, as many native Chinese plants rely on it for pollination. Some DnaJ genes, such as *DnaJA1*, *DnaJB6*, *DnaJB12*, *DnaJB13*, *DnaJB14*, and *DnaJC8*, are key to the stress response of *A. cerana cerana*. In this study, we aimed to elucidate the characteristics of *DnaJC28* in *A. cerana cerana* and to determine whether it mediates defense against environmental stress in honeybees.

## Materials and methods

### Identification of the *AccDnaJC28* gene coding sequence

The coding sequence (CDS) of *AccDnaJC28* was amplified using polymerase chain reaction (PCR) with gene-specific primers (**Supplementary Table S1**), which were designed on the basis of the *Apis cerana DnaJC28* CDS (gene symbol: LOC108001060). Amplification was performed using TransFast^®^ Taq DNA Polymerase (TransGen Biotech, China) in accordance with the manufacturer’s instructions.

### Bioinformatics analysis of *AccDnaJC28*

The J domains of AccDnaJC28 were predicted using the Pfam protein family database. Multiple alignments of the amino acid sequences were performed using DNAMAN. A phylogenetic tree was constructed using the neighbor-joining algorithm in MEGA. This distance-based method follows the minimum evolution principle and does not depend on optimization criteria. We obtained the amino acid sequence of DnaJC28 from the NCBI database. The details of the various DnaJC28 proteins are provided in **Supplementary Table S2**. The tertiary structure of the AccDnaJC28 J domain was initially modeled in SWISS-MODEL, and further analysis was conducted in SPDBV.

### Analysis of the subcellular localization of AccDnaJC28

Subcellular localization was analyzed via standard protocols, as described previously (Yang et al., 2013; Zhou et al., 2015; Li et al., 2020b). The CDS of AccDnaJC28 was cloned and inserted into the pUAST-6×Myc vector to construct the recombinant plasmid pUAST-6×Myc-DnaJC28 using the primers listed in **Supplementary Table S1**. *Drosophila* S2 cells were cultured at 25 °C in Schneider’s Drosophila Medium (Invitrogen, USA) supplemented with 10% fetal bovine serum (Gibco, USA), 1% penicillin, and 1% streptomycin (Sangon Biotech, China). The recombinant plasmid pUAST-6×Myc-DnaJC28 was transfected using polyetherimide (Sigma, Germany) according to the manufacturer’s protocol. At 48 h post-transfection, the cells were harvested, washed with PBS for 10 min, and fixed with 4% formaldehyde (diluted in PBS) for 20 min. After fixation, the cells were washed three times with PBS (10 min per wash). The cells were subsequently incubated overnight with an anti-Myc antibody (Santa Cruz, USA). The following day, the cells were washed with PBS and incubated for 2 h in the dark with a Cy3-conjugated secondary antibody (Jackson ImmunoResearch, USA), followed by a 15-min incubation with 4′,6-diamidino-2-phenylindole (DAPI; Santa Cruz, USA) in the dark. Finally, the cells were washed again with PBS. Images were acquired with a ZEISS LSM880 ultrahigh-resolution laser scanning microscope.

### Honeybee materials and treatments

*A. cerana cerana* colonies were maintained at Qufu Normal University (Qufu, China) for breeding purposes. Three colonies (queenright and healthy), which represented three independent biological replicates, were used. The sampling period for *A. cerana cerana* was from June to September. *A. cerana cerana* individuals were categorized according to their developmental stage as larvae (aged one to seven days), prepupae, pupae (subdivided into white-eyed, pink-eyed, brown-eyed, and dark-eyed pupae), and adult workers. Samples of one-day-old workers, pupae, prepupae and larvae were collected directly from the hive. For 19-day-old workers, individuals were marked with paint on their first day after emergence and then retrieved from outdoor hives 19 days later to perform different stress treatments and to obtain specific tissues. Various tissues, including the head, thorax, abdomen, stinger, midgut, epidermis, muscle, antennae, legs, and rectum, were dissected on an ice-cooled surface.

Nineteen-day-old workers were randomly divided into 12 groups (n=50 per group). Honeybees in groups 1–4 were placed in incubators set at 4 °C, 40 °C, 43 °C, and 46 °C, respectively. Honeybees in groups 5, 6, and 7 were fed 1 µL of paraquat (2 mg/mL), beta-cypermethrin (0.05 mg/mL), or abamectin (0.05 mg/mL), respectively, by using an Eppendorf pipette with a measuring range of 0.1–2.5 µL; the target doses were prepared by dissolution or dilution with water according to the manufacturers’ instructions. Each honeybee in group 8 was fed 1 µL of 2 mM H_2_O_2_ by using an Eppendorf pipette with a measuring range of 0.1–2.5 µL. Honeybees in group 9 were exposed to UV stress (30 mJ/cm²). A UV-C low-pressure mercury lamp with a main emission peak at 254 nm was used for UV treatment, and the irradiation time required to achieve 30 mJ/cm² was 30 s. Group 10 served as the untreated control for groups 1–9. All the honeybees from groups 5–10 were incubated at 33 °C. Honeybees from groups 1–10 were sampled at 0, 1, 2, 3, 4, and 5 h after treatment. Honeybees in group 11 were placed in an incubator at 40 °C and sampled at 0, 12, 24, 36, and 48 h, whereas those in group 12, which were incubated at 33 °C, served as the controls for those in group 11. Honeybees in all groups were maintained in the dark at 70% relative humidity and provided a 50% sucrose solution ad libitum. All the samples from all the groups included three *A. cerana cerana* individuals.

### RNA extraction, cDNA synthesis and reverse transcription quantitative PCR

Total RNA was isolated with TRIzol reagent (TaKaRa, China) in accordance with the manufacturer’s instructions. cDNA was subsequently synthesized by reverse transcription using HiScript^®^ II Q RT SuperMix for qPCR (+ gDNA Wiper) (TaKaRa, China), according to the manufacturer’s protocol. Reverse transcription–quantitative PCR (RT–qPCR) was performed using an SYBR^®^ Green Premix Pro Taq HS qPCR Kit (Accurate Biotechnology, Hunan, China) according to the manufacturer’s instructions. RT–qPCR amplification was performed using a CFX96 Real-Time Detection System (Bio-Rad, USA) with the primers listed in **Supplementary Table S1**. All primers were designed following established RT–qPCR guidelines and validated to meet the following criteria: a single peak in the melting curve analysis, amplification efficiency (E) ranging from 90% to 110%, and a correlation coefficient (R²) between 0.98 and 1.00. Gene expression levels, which were normalized to the expression levels of the reference gene *β-actin*, were determined via the 2^-ΔΔCT^ method. The *β-actin* gene was selected as a reference gene because it is stably expressed across different honeybee tissues and under various stress conditions and is commonly used in gene expression studies, including RNAi experiments in honeybees (Amdam et al., 2004; Li et al., 2012; Wang et al., 2012; Li et al., 2020b). Three independent biological replicates were used for RT–qPCR, and three technical replicates were run for each corresponding cDNA sample.

### RNA interference assay

The partial CDS of AccDnaJC28 (170–670 nt) was amplified from *A. cerana cerana* cDNA using PCR with primers (**Supplementary Table S1**) containing the T7 RNA polymerase promoter sequence added at the 5’ end. This amplified sequence was subsequently used to synthesize AccDnaJC28 double-stranded DNA (dsRNA-DnaJC28) using the T7 RiboMAX™ Express RNA Interference (RNAi) System (Promega, USA) following the manufacturer’s protocol. dsRNA targeting *GFP* (dsRNA-GFP; GenBank: U87974.1) was also generated and used as a control for dsRNA-DnaJC28.

Forty 19-day-old workers were equally divided into two groups. Each worker in the treatment group received 5 µg of dsRNA-DnaJC28, whereas each worker in the control group received 5 µg of dsRNA-GFP. All workers were then maintained at 33 °C in constant darkness and 70% relative humidity. RNAi efficiency was assessed via RT–qPCR after 24, 48, and 72 h of feeding. Three healthy honeybee colonies were used in the RNAi experiments, and each colony represented an independent biological replicate. Furthermore, three *A. cerana cerana* individuals were included in each sample.

### Evaluation of the heat resistance of honeybees after *AccDnaJC28* silencing

Nineteen-day-old workers were assigned to two groups. Honeybees in group 1 were administered 5 µg of dsRNA-DnaJC28, while those in group 2 received 5 µg of dsRNA-GFP. Afterward, the honeybees in both groups were subjected to heat tolerance testing at 43 °C, and their survival rates were recorded at 0.5 h intervals. In this experiment, three healthy honeybee colonies were used, and each represented an independent biological replicate. Additionally, three *A. cerana cerana* individuals were included in each sample.

### Changes in the expression of antioxidant genes following *AccDnaJC28* knockdown

RT–qPCR was used to assess the expression of *CYP4G11*, *CDK5*, *CDK5r*, *Trx1*, *Trx2*, *Tpx4*, *Tpx5*, *Hsp22.6*, *MsrB*, *GSTO2*, *GSTS4*, *GSTD*, *GSTT1*, *SOD1*, *MKK4*, *CYP336A1*, *GSTZ1*, *TrxR1*, *p38b*, and *Tpx3* after RNAi-mediated knockdown of *AccDnaJC28* using the primers listed in **Supplementary Table S1**. Earlier findings from our laboratory indicate that these genes play a role in the antioxidant defense system.

### Effects of *AccDnaJC28* silencing on the activity of antioxidant enzymes

For the antioxidant enzyme assays, a 10% whole-honeybee homogenate was prepared in ice-cold saline. The total protein content in the homogenate was first quantified using a commercial Coomassie brilliant blue kit according to the manufacturer’s protocol. Catalase (CAT), peroxidase (POD), and total superoxide dismutase (T-SOD) enzyme activities were measured using a CAT assay kit (serial number: A007-1-1), POD assay kit (serial number: A084-1-1), and T-SOD assay kit (serial number: A001-1-1), respectively, according to the manufacturer’s instructions. These test kits were purchased from the Nanjing Jiancheng Bioengineering Institute (Nanjing, China). Enzyme activity is presented in U/mgprot, with “prot” as the abbreviation for “protein”. Three healthy honeybee colonies, each constituting an independent biological replicate, were used in this experiment. In addition, each sample consisted of three *A. cerana cerana* individuals.

### Evaluation of the thermotolerance of *Drosophila* S2 cells upon *AccDnaJC28* overexpression

The recombinant plasmid pUAST-6×Myc-DnaJC28 was transfected into *Drosophila* S2 cells, while cells in the control group were transfected with the vector pUAST-6×Myc. After being cultured at 25 °C in an aseptic incubator for 48 h, the transfected cells were transferred to a 37 °C aseptic incubator for heat stress treatment. Cell status was evaluated using an inverted white light/fluorescence microscope at 0, 18, and 48 h post-treatment. Additionally, at 48 h post-treatment, cell viability was assessed in both groups using a trypan blue staining kit (Beyotime, China). Following staining, images were captured with an upright white light microscope to evaluate cell morphology. A hemocytometer was then used to count the dead and total cells to calculate the cell mortality rate.

### Statistical analysis

All data were obtained from three replicates. For comparisons of two independent values, Student’s *t* tests were performed in SAS (version 9.1), and **P < 0.01 was considered to indicate statistical significance. For comparisons involving multiple groups, one-way ANOVA followed by Tukey’s *post hoc* test was conducted in SPSS (version 22) (*P* < 0.05; distinct letters indicate significant differences).

## Results

### Sequence analysis and evolutionary relationships of AccDnaJC28

The CDS of *AccDnaJC28* is 1125 bp and encodes a protein of 374 amino acids. To examine the evolutionary relationships between DnaJC28 proteins and DnaJ proteins from other animals, we conducted a phylogenetic analysis. As shown in **Figure 1**, AccDnaJC28 was more closely related to the DnaJC subfamily than to the DnaJA and DnaJB subfamilies and was more homologous to other animal DnaJC28 proteins, especially *Apis mellifera* DnaJC28 (AmDnaJC28). Multiple amino acid sequence alignments revealed that AccDnaJC28 exhibited greater sequence similarity to AmDnaJC28 than to other animal DnaJC28 proteins, which is consistent with the results of the phylogenetic tree analysis (**Supplementary Figure S1**).

**Figure 1:**
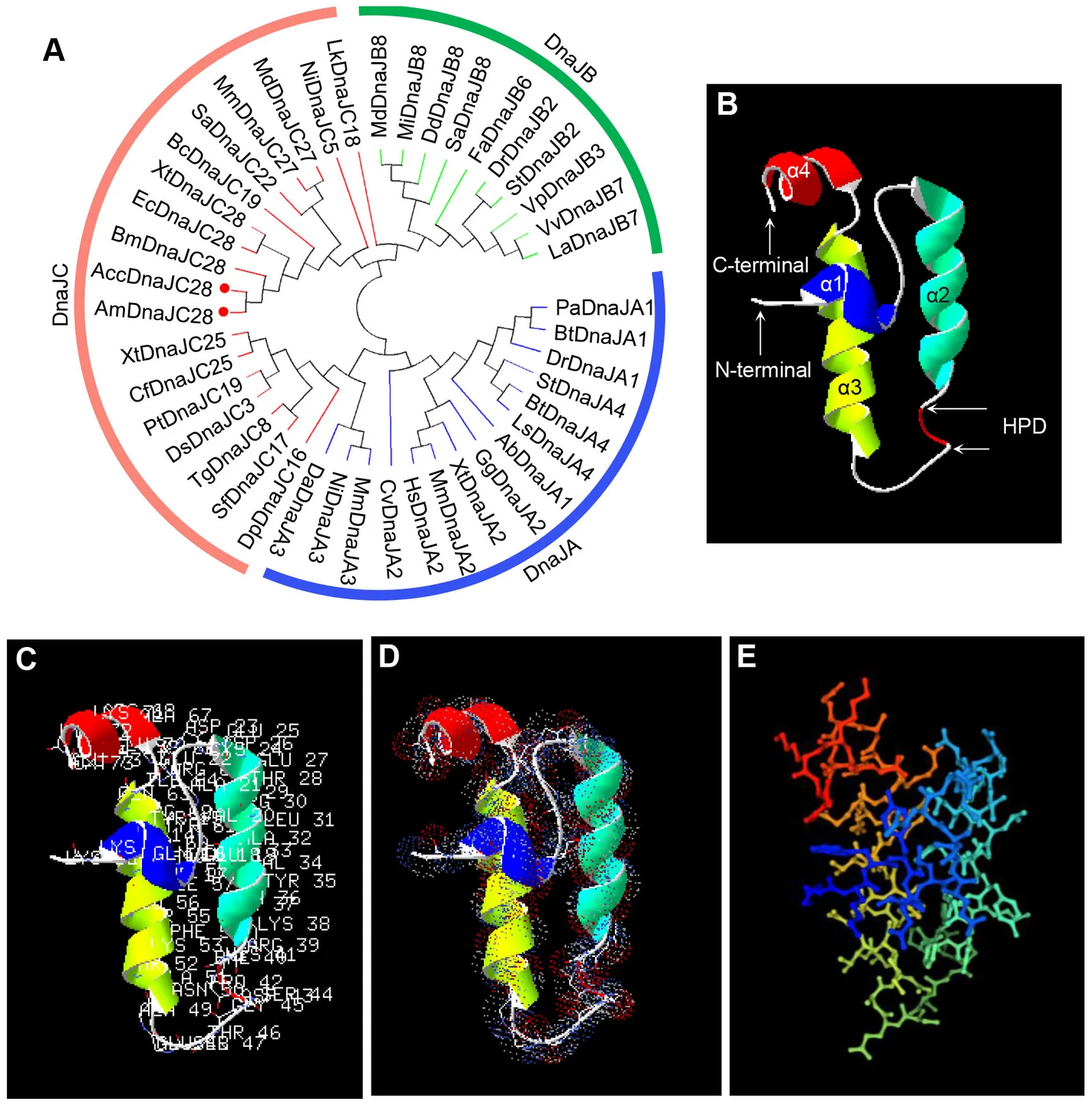
Phylogenetic tree of AccDnaJC28 and analysis of the tertiary structure of its J domain. **(A)** Phylogenetic tree analysis of AccDnaJC28. AccDnaJC28 and AmDnaJC28 are indicated with red circles. The amino acid sequences of DnaJC28 were retrieved from the NCBI online database, and detailed descriptions of different DnaJC28 proteins are provided in **Supplementary Table S2**. **(B)** Tertiary structure of the AccDnaJC28 J domain. **(C–E)** Amino acid residues, electrostatic surface distribution, and hyperball models of the AccDnaJC28 J domain.

### AccDnaJC28 has a typical J domain

To determine the precise amino acid sequence and location of the J domain in AccDnaJC28, we used the Pfam protein family database. The J domain of AccDnaJC28 is located between amino acids 41 and 125 and comprises 85 amino acids. Tertiary structure prediction revealed that the J domain of AccDnaJC28 is composed of four α-helices (helices I–IV), with the characteristic His, Pro, and Asp (HPD) tripeptide located between helices II and III (**Figures 1B–E**). As shown in **Figures 1C, D**, the J domain of AccDnaJC28 contains 15 surface-exposed negatively charged amino acids (4 aspartic acid (Asp/D) residues and 11 glutamic acid (Glu/E) residues), 14 surface-exposed positively charged amino acids (7 lysine (Lys/K) residues, 5 arginine (Arg/R) residues, and 2 histidine (His/H) residues), and 26 surface-exposed uncharged hydrophobic amino acids (1 glycine (Gly/G) residue, 7 alanine (Ala/A) residues, 3 valine (Val/V) residues, 4 leucine (Leu/L) residues, 4 isoleucine (Ile/I) residues, 2 proline (Pro/P) residues, 4 phenylalanine (Phe/F) residues, and 1 tryptophan (Trp/W) residue).

### AccDnaJC28 is located in the cytoplasm

Owing to the difficulty in culturing honeybee cells, we chose to use *Drosophila* S2 cells for subcellular localization analysis. The CDS of AccDnaJC28 was inserted into the pUAST-6×Myc plasmid and subsequently transfected into *Drosophila* S2 cells for expression, followed by immunostaining with an anti-Myc antibody. As shown in **Figure 2A**, AccDnaJC28 was located in the cytoplasm, indicating that AccDnaJC28 plays a role in the cytoplasm.

**Figure 2:**
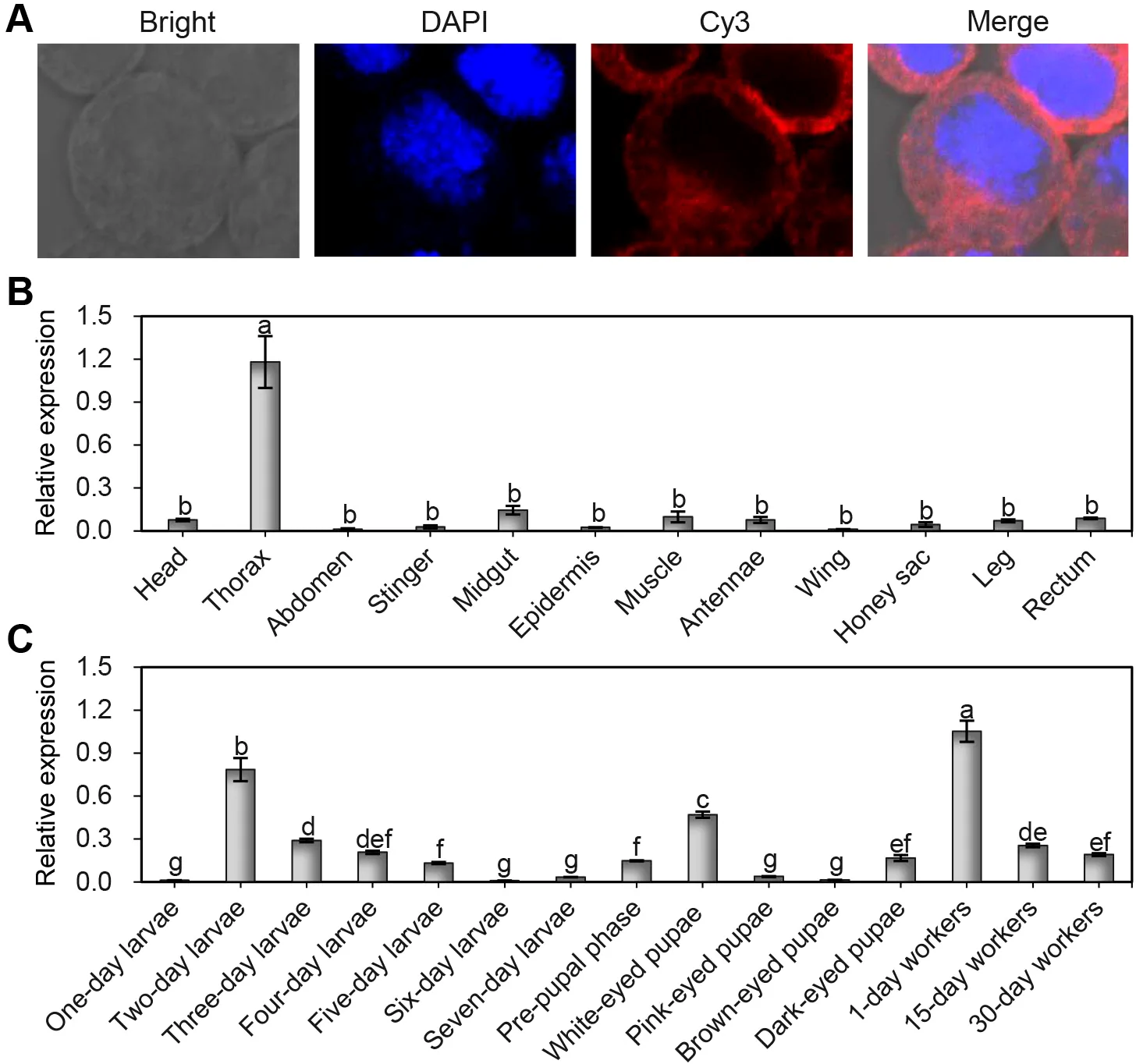
Subcellular localization of AccDnaJC28 and spatiotemporal specificity of its expression. **(A)** Subcellular localization analysis of AccDnaJC28. Cy3 (red) was used to label AccDnaJC28, and DAPI (blue) was used to stain the cell nuclei. **(B, C)** AccDnaJC28 expression across various tissues **(B)** and developmental stages **(C)** was analyzed, with *β-actin* serving as the loading control. The data are shown as the mean ± SE of three replicates. Statistically significant differences are indicated by different letters (Tukey’s *post hoc* test, *P* < 0.05).

### *AccDnaJC28* exhibits spatiotemporal-specific expression

To assess the expression patterns of *AccDnaJC28* in various tissues and at different developmental stages, we conducted RT–qPCR assays. As shown in **Figure 2B**, *AccDnaJC28* was broadly expressed across various tissues, including the head, abdomen, stinger, midgut, epidermis, muscle, antennae, legs, and rectum, with the thorax showing the highest expression levels. In terms of developmental stages, *AccDnaJC28* expression peaked in 1-day-old worker bees and was moderately greater in two-day-old larvae and white-eyed pupae than in bees at the other developmental stages; statistically significant differences according to Tukey’s *post hoc* test (*P* < 0.05) are indicated by different letters (**Figure 2C**). These results suggest that *AccDnaJC28* may play a key role in the growth and development of *A. cerana cerana*.

### *AccDnaJC28* plays an important role under different degrees of heat stress

Given that *AccDnaJC28* is a member of the Hsp gene family, we first focused on its response to heat stress. We established gradients for the degree and duration of heat treatment. As shown in **Figure 3A**, the mRNA levels of *AccDnaJC28* did not significantly change in honeybees exposed to 33 °C for 5 h. When *A. cerana cerana* honeybees were exposed to 40, 43, and 46 °C for 5 h, the expression level of *AccDnaJC28* increased, and *AccDnaJC28* expression was greatest after 2, 5, and 4 h of exposure to these temperatures, respectively (**Figures 3B–D**). The duration of heat treatment was extended to 48 h to further investigate the role of *AccDnaJC28*, and the results revealed that its expression remained stable at 33 °C (**Figure 3E**). However, *AccDnaJC28* expression was markedly upregulated at 40 °C from 12 to 48 h (**Figure 3F**). Statistically significant differences according to Tukey’s *post hoc* test (*P* < 0.05) are denoted by different letters (**Figure 3**). These results indicate that the heat stress response of *A. cerana cerana* may be mediated, at least in part, by the increased expression of *AccDnaJC28*.

**Figure 3:**
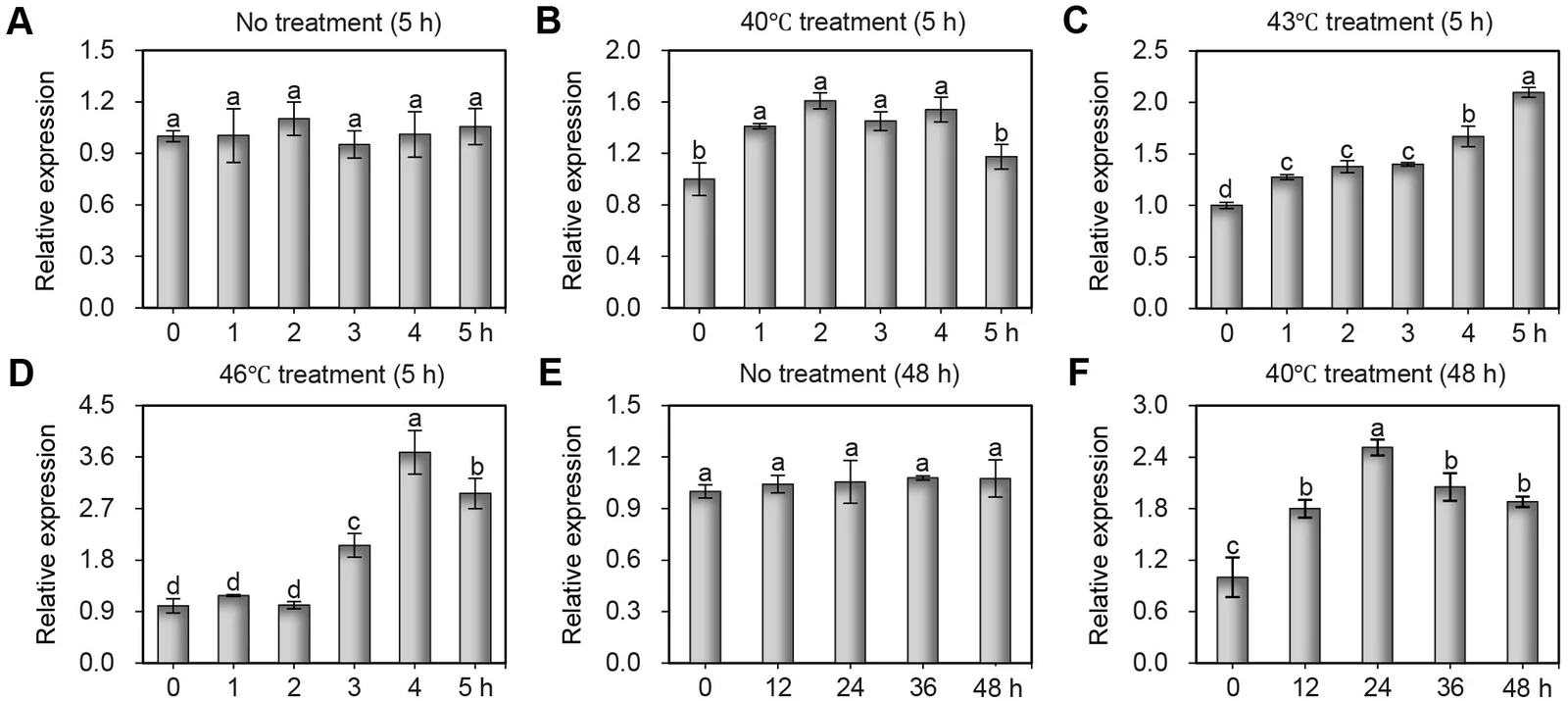
mRNA level of *AccDnaJC28* under heat stress conditions. The expression level of *AccDnaJC28* after exposure to 40 °C **(B)**, 43 °C **(C)**, and 46 °C **(D)** for 5 h and after exposure to 40 °C for 48 h **(F)** normalized to the level at 33 °C (A and E represent no treatment for 5 and 48 h, respectively). *β-actin* served as the internal reference gene. The values are presented as the mean ± SE of three replicates. Different letters denote statistically significant differences according to Tukey’s *post hoc* test (*P* < 0.05).

### *AccDnaJC28* transcription is induced by other stresses

To evaluate the response of *AccDnaJC28* to stress factors other than heat stress, RT–qPCR analysis was conducted. Under cold stress conditions, the mRNA level of *AccDnaJC28* increased sharply at 1 h but then partially decreased from 2 to 3 h before finally returning to control levels by 4–5 h (**Figure 4A**). The expression level of *AccDnaJC28* increased from 3 to 5 h under both H_2_O_2_ exposure and UV stress, peaking at 3 h after H_2_O_2_ exposure and at 4 h after UV stress exposure (**Figures 4B, C**). In addition, the transcription level of *AccDnaJC28* increased when *A. cerana cerana* was treated with pesticides, reaching maximum levels at 2, 1, and 2 h after treatment with paraquat, beta-cypermethrin, and abamectin, respectively (**Figures 4D–F**). Different letters represent statistically significant differences according to Tukey’s *post hoc* test (*P* < 0.05) (**Figure 4**). These results indicate that cold exposure, H_2_O_2_ exposure, UV exposure, and exposure to different pesticides can induce the expression of *AccDnaJC28* to varying degrees. The differential induction of *AccDnaJC28* expression under various stress conditions implies that *AccDnaJC28* expression in *A. cerana cerana* is modulated in response to specific environmental challenges.

**Figure 4:**
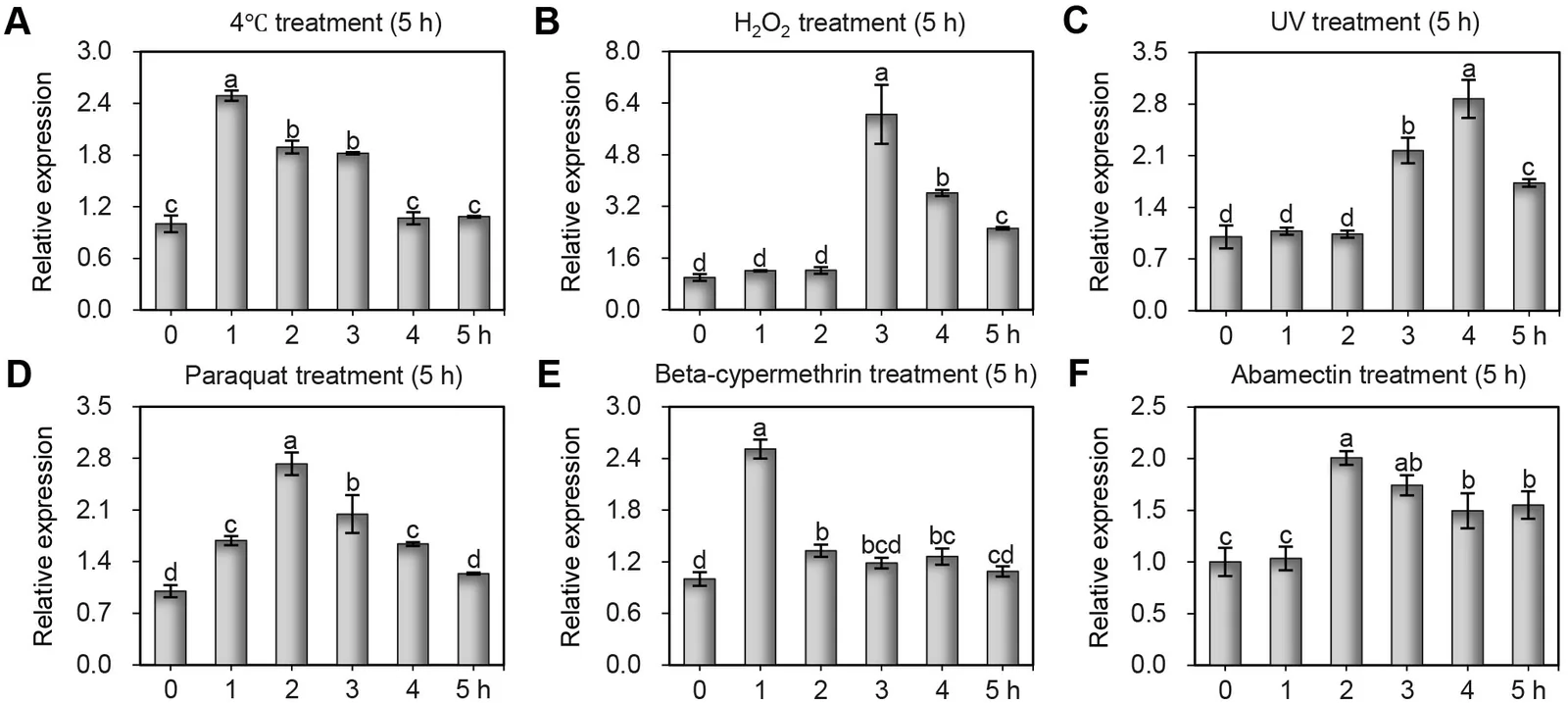
Expression level of *AccDnaJC28* under various stress conditions. Transcript levels of *AccDnaJC28* in *A. cerana cerana* following exposures to 4 °C **(A)**, H_2_O_2_ **(B)**, UV **(C)**, paraquat **(D)**, beta-cypermethrin **(E)**, and abamectin **(F)**. *β-actin* served as the internal reference. Each value represents the mean ± SE of three replicates. Different letters indicate significant differences according to Tukey’s *post hoc* test (*P* < 0.05).

### RNAi-mediated *AccDnaJC28* knockdown decreases the heat resistance of *A. cerana cerana*

To further explore the function of *AccDnaJC28* under stress conditions, we first knocked down its expression in *A. cerana cerana* by feeding honeybees with dsRNA targeting *AccDnaJC28*. As shown in **Figure 5A**, significant transcriptional suppression was observed in the dsRNA-DnaJC28-fed group compared with that in the dsRNA-GFP-fed control group, indicating that *AccDnaJC28* silencing was successful. In addition, the silencing efficiency at 24 h after dsRNA-DnaJC28 administration was greater than that at 48 h and 72 h after dsRNA-DnaJC28 administration, with a knockdown efficiency of 86.95%. Therefore, *A. cerana cerana* honeybees fed dsRNA-DnaJC28 for 24 h were selected for subsequent analyses, including thermotolerance analysis, antioxidant gene expression detection, and enzyme activity assays.

**Figure 5:**
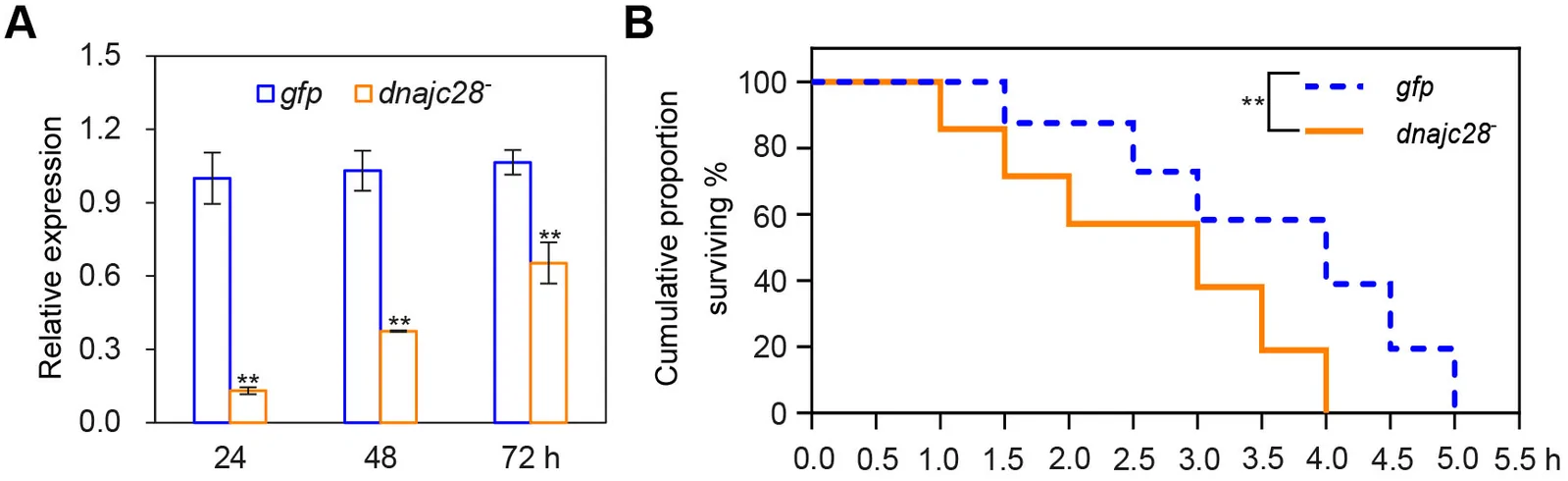
Measurement of the efficiency of *AccDnaJC28* knockdown **(A)** and assessment of the effect of *AccDnaJC28* silencing on honeybee thermotolerance **(B)**. **(A)** Knockdown efficiency was measured by RT–qPCR using *β-actin* as an internal control, and data from three replicates are presented. The groups are abbreviated “*dnajc28*^-^” (*AccDnaJC28* knockdown) and “*gfp*” (GFP control). **(B)** The survival status of the honeybees was recorded every 0.5 h. Statistical significance is indicated by **P < 0.01.

We subsequently subjected *A. cerana cerana* to heat treatment and found that compared with the control treatment, *AccDnaJC28* knockdown significantly reduced the survival rate of *A. cerana cerana* under heat stress conditions (**Figure 5B**). We hypothesized that the overexpression of *AccDnaJC28* could increase the heat tolerance of *A. cerana cerana*.

### Overexpression of AccDnaJC28 increases the heat tolerance of *Drosophila* S2 cells

Currently, it is not feasible to apply transgenic technologies to honeybees. Therefore, we used *Drosophila* S2 cells that heterologously overexpressed *AccDnaJC28* to further investigate the function of AccDnaJC28 in heat resistance. Heterologous overexpression of *AccDnaJC28* in *Drosophila* S2 cells markedly increased cellular density and viability following exposure to heat stress, with the effect being particularly pronounced after 48 h (**Figure 6A**). Trypan blue staining revealed that the percentage of dead *Drosophila* S2 cells was notably lower in the *AccDnaJC28* overexpression group than in the control group after 48 h of heat stress (**Figures 6B, C**). These results suggest that *AccDnaJC28* plays a critical role in *A. cerana cerana* under heat stress, with its overexpression increasing the heat tolerance.

**Figure 6:**
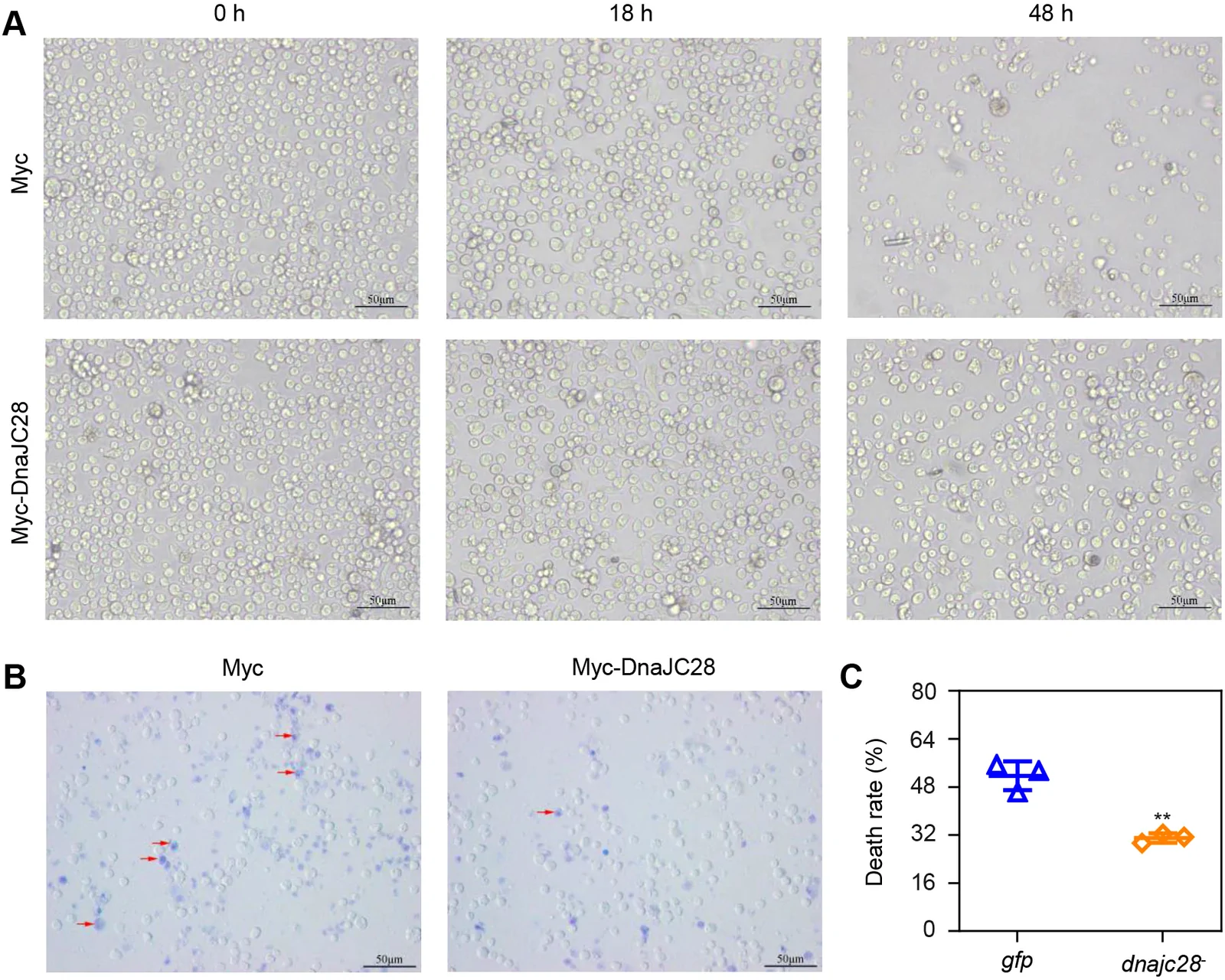
Effects of *AccDnaJC28* on the heat resistance of *Drosophila* S2 cells. **(A)** The state of *Drosophila* S2 cells after heat treatment for 0 h, 18 h and 48 h. *AccDnaJC28* was cloned and inserted into the pUAST-6×Myc vector (Myc-DnaJC28), and this vector was subsequently transfected into *Drosophila* S2 cells prior to exposure to heat stress. *Drosophila* S2 cells transfected with the pUAST-6×Myc empty vector (Myc) were used as controls. **(B)** Trypan blue staining of *Drosophila* S2 cells after heat treatment for 48 h. Dead cells were stained blue. Some of the dead and stained cells are marked with red arrows. **(C)** Comparison of cell death rates between AccDnaJC28-overexpressing and control *Drosophila* S2 cells after 48 h of heat stress. Data from three replicates are shown. **P < 0.01 (Student’s *t* test).

### *AccDnaJC28* knockdown influences the expression of antioxidant-related enzymes and genes

To investigate the mechanism of action of *AccDnaJC28* in the stress response, we investigated how silencing *AccDnaJC28* affected the expression of antioxidant-related enzymes and genes. As shown in **Figures 7A–O**, *AccDnaJC28* RNAi increased the expression levels of *CYP4G11*, *CDK5*, *CDK5r*, *Trx1*, *Trx2*, *Tpx4*, *Tpx5*, *Hsp22.6*, *MsrB*, *GSTO2*, *GSTS4*, *GSTD*, *GSTT1*, *SOD1*, and *MKK4*. In contrast, *AccDnaJC28* knockdown decreased the mRNA levels of *CYP336A1*, *GSTZ1*, and *TrxR1* (**Figures 7P–R**) but did not significantly affect the expression levels of *p38b* or *Tpx3* (**Figures 7S, T**). Moreover, silencing *AccDnaJC28* led to a significant decrease in the enzymatic activities of POD and T-SOD, but no significant change in CAT activity was observed (**Figure 8**). These findings suggest that *AccDnaJC28* knockdown may perturb the activity of the antioxidant signaling pathway in *A. cerana cerana*, with concomitant upregulation of the expression of multiple antioxidant genes involved in functional compensation.

**Figure 7:**
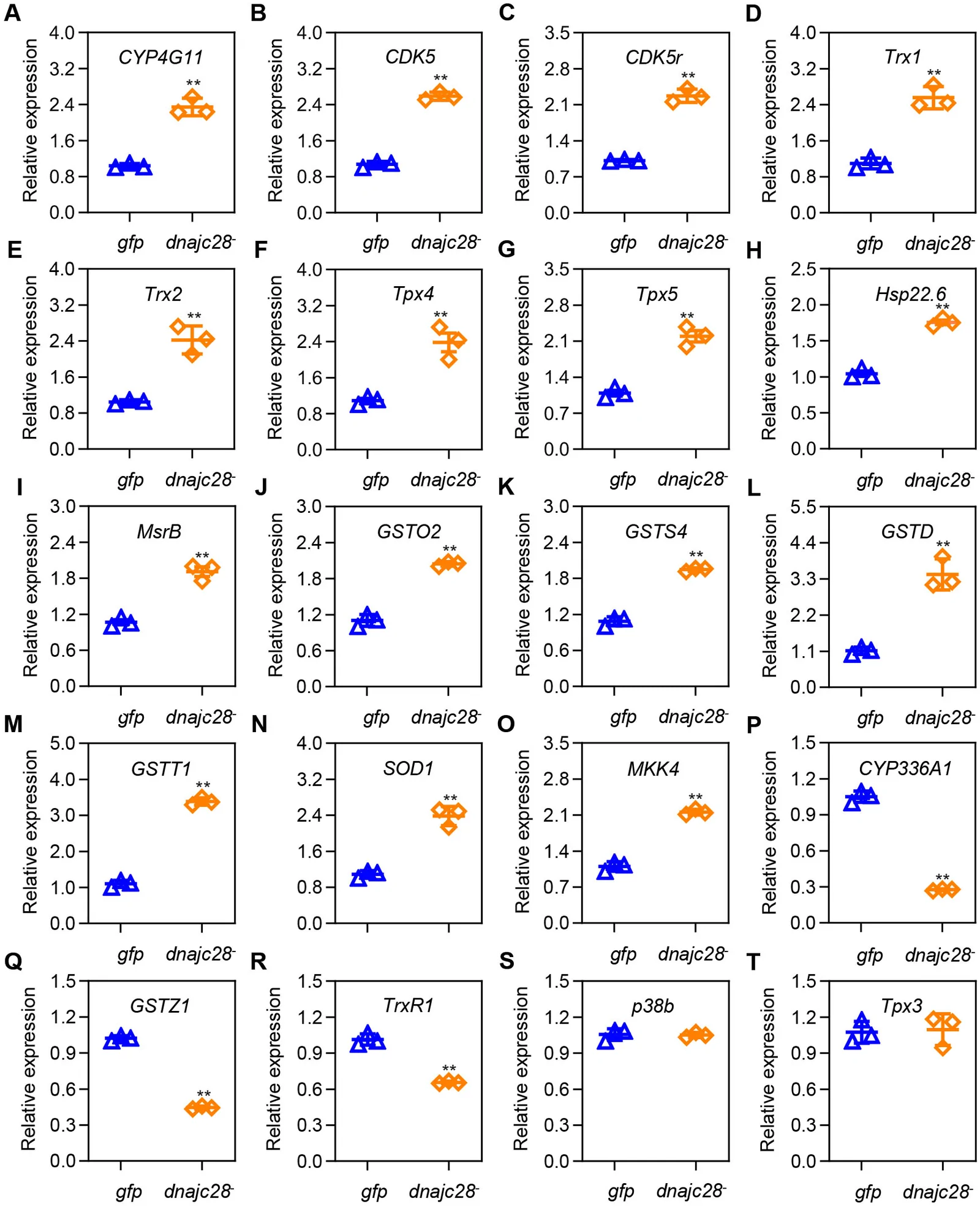
Effect of *AccDnaJC28* silencing on the expression levels of antioxidant genes. **(A–T)** Expression levels of *CYP4G11*, *CDK5*, *CDK5r*, *Trx1*, *Trx2*, *Tpx4*, *Tpx5*, *Hsp22.6*, *MsrB*, *GSTO2*, *GSTS4*, *GSTD*, *GSTT1*, *SOD1*, *MKK4*, *CYP336A1*, *GSTZ1*, *TrxR1*, *p38b* and *Tpx3*. **P < 0.01 indicates a significant difference. The designations “*dnajc28*^-^” and “*gfp*” indicate the *AccDnaJC28* knockdown and GFP control groups, respectively.

**Figure 8:**
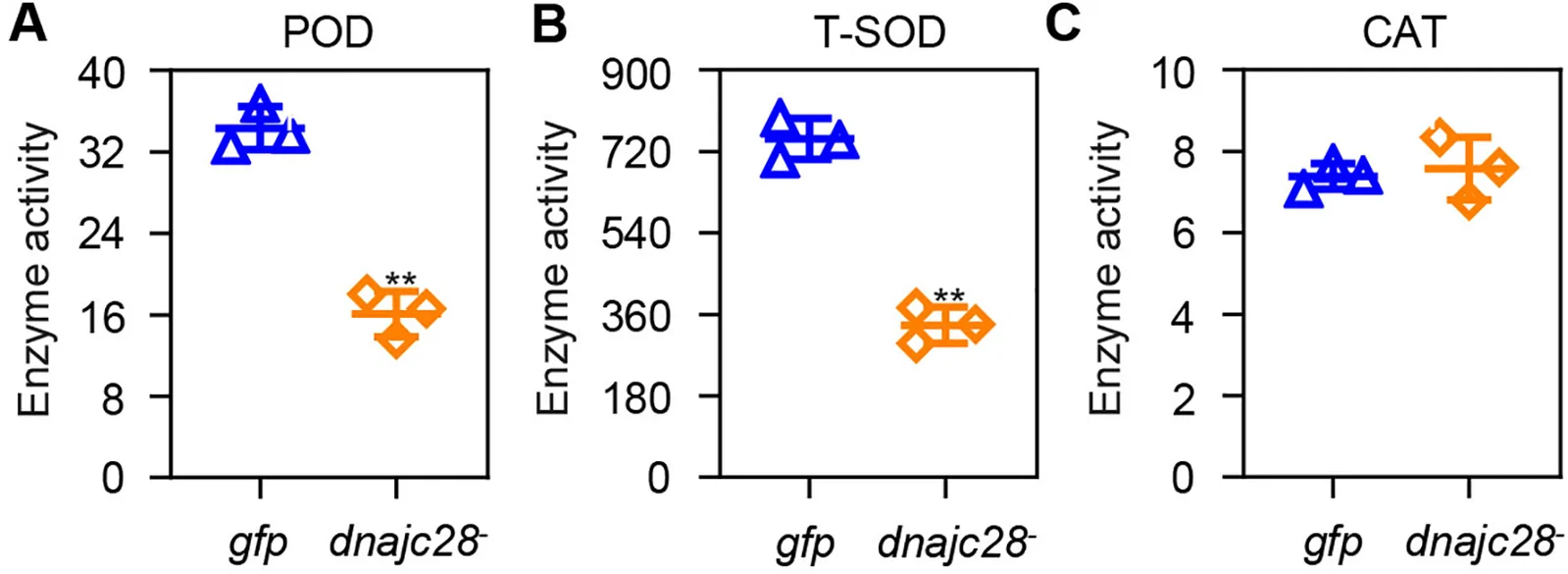
Enzyme activities of POD, T-SOD, and CAT after *AccDnaJC28* knockdown. Enzyme activities of **(A)** POD, **(B)** T-SOD, and **(C)** CAT after *AccDnaJC28* knockdown. Groups: *dnajc28*^-^ (*AccDnaJC28* knockdown) and *gfp* (control). Data from three replicates are presented. **P < 0.01 indicates a significant difference (Student’s *t* test).

## Discussion

Honeybees are key pollinators that sustain both natural and agricultural ecosystems. However, their health is often threatened by various environmental stresses (Klein et al., 2007; Potts et al., 2010; Goulson et al., 2015; Farias et al., 2023; Requier et al., 2023). Therefore, elucidating the mechanisms through which honeybees adapt to environmental stressors is vital for devising strategies to protect the health of honeybee colonies. In this study, we found that AccDnaJC28 is a DnaJ protein located in the cytoplasm and that the induction of *AccDnaJC28* expression is vital for honeybees to resist environmental stress.

The DnaJ protein is characterized by a conserved J domain consisting of four α-helices, with an HPD tripeptide situated in an exposed loop region of this domain (Jiang et al., 2007; Kampinga and Craig, 2010). We found that AccDnaJC28 possesses a J domain that features four α-helices and an HPD tripeptide (**Figures 1B–G**), indicating that AccDnaJC28 is a typical DnaJ protein. DnaJ proteins have been shown to localize to diverse cellular compartments. A previous study revealed that upon fractionation of HeLa cells, DnaJ was primarily cytoplasmic, with low levels detected in the microsomal fraction (Ohtsuka et al., 1993). *Leishmania* DnaJ, on the basis of its putative orthologs and structural features, may be localized to various subcellular compartments, including the mitochondria, endoplasmic reticulum, flagellar pockets, endosomes, and nucleus (Diefenbach and Kindl, 2000). DnaJA1 and DnaJB12 have been reported to be located in the cytoplasm, whereas DnaJC8 is located in the nucleus in *A. cerana cerana* (Li et al., 2020a). We found that AccDnaJC28 was located in the cytoplasm (**Figure 2A**), indicating that AccDnaJC28 functions in the cytoplasm. To our knowledge, this is the first study to experimentally determine the subcellular localization of DnaJC28 in animals, and the results provide a theoretical foundation for investigating the subcellular localization and nuclear functions of DnaJC28 in other animal species.

Previous studies have shown that *Hsps* expression is spatiotemporally specific and varies among different developmental stages in different species. For example, the expression level of *Hsp40* remains relatively constant during the larval phase but significantly increases during both the pupal and adult stages in *Cotesia vestalis* (Shi et al., 2013). During the cleavage, blastocyst, neuroembryonic, and tail bud stages in *Thamnaconus septentrionalis*, *Hsp90ab1* expression peaks, whereas*Hsp8.2* expression is minimal (Chen et al., 2024). In *Liriomyza sativae*, *sHsp* expression is highest during the pupal phase, whereas *Hsp60* and *Hsp90* mRNA levels increase progressively throughout development (Huang et al., 2009). We observed that although the expression of *AccDnaJC28* varied across different developmental stages of *A. cerana cerana*, it remained detectable throughout multiple stages (**Figure 2C**). Some DnaJ proteins play crucial roles in the stress response and, as Hsp70 cochaperones, are important for protein unfolding, folding, translation, translocation, and degradation (Shi et al., 2006; Roh et al., 2008; Craig and Marszalek, 2017; Li et al., 2018; Chand et al., 2021; Wang et al., 2025). The dynamic, stage-specific expression of *AccDnaJC28* may reflect its roles in protein homeostasis and stress responses and likely corresponds to the physiological demands and challenges of each developmental stage in *A. cerana cerana*.

According to previous studies, Hsps also display distinct spatiotemporal expression patterns among different tissues. For example, *DnaJA1* is expressed primarily in the testes, and its knockout results in complete male infertility in mice (Mao et al., 2024). DnaJ1 proteins are most abundant in hemocytes, with lower levels detected in the fat body and head of *Bombyx mori* (Li et al., 2016). In Chinese Holstein cattle, the highest level of *HSP70A1A* mRNA expression was observed in the heart, with progressively lower levels in the muscle and liver (Li et al., 2011). We found that *AccDnaJC28* expression was higher in the thorax than in all the other tissues examined (**Figure 2B**). The thorax temperature of honeybees can be regulated under cold and heat stresses (Heinrich, 1979; Coelho, 1991; Kleinhenz et al., 2003) and is directly correlated with flight performance and foraging activity (Woods et al., 2005; Kovac et al., 2010; Sadler and Nieh, 2011). The high expression level of *AccDnaJC28* in the thorax may contribute to the regulation of thorax temperature in *A. cerana cerana* and thereby enhance flight performance and increase foraging activity in response to cold and heat stresses.

*DnaJC28* is a key gene involved in thermoregulation in Chinese rhesus macaques and in tropical adaptation in Nellore cattle (Fernandes Junior et al., 2020; Wang et al., 2025). We found that *AccDnaJC28* expression was induced by cold and heat stress (**Figures 3**, **4A**). Functional analysis further revealed that compared with dsRNA-GFP (control) treatment, RNAi-mediated *AccDnaJC28* knockdown with dsRNA-DnaJC28 reduced survival rates in *A. cerana cerana* under heat stress (**Figure 5B**), whereas *AccDnaJC28* overexpression promoted survival in *Drosophila* S2 cells exposed to similar conditions (**Figure 6**). Cold stress impairs honeybee development and increases misorientation and pupal and adult mortality (Wang et al., 2016), whereas heat stress alters immunity, forewing asymmetry, longevity, and foraging onset age in honeybees (Medina et al., 2020). On the basis of these findings and those of our study, we propose that *AccDnaJC28* is a crucial gene for thermoregulation and that its overexpression enhances the temperature resistance of *A. cerana cerana* under both cold and heat stresses.

Notably, the design of the *in vivo* RNAi experiment had several limitations. Ideally, an untreated/vehicle control group should have been included alongside the dsRNA-GFP control to exclude any nonspecific effects caused by dsRNA administration itself. However, in our experimental group (fed dsRNA-DnaJC28) and the control group (fed dsRNA-GFP), only one variable was manipulated: apart from the dsRNA administered, all the other experimental conditions were identical. Therefore, the only intended difference between the experimental and control groups in the RNAi experiment was the dsRNA sequence, which allowed the observed phenotype to be attributed to the sequence-specific knockdown of *AccDnaJC28*.

In the present study, we found that UV stress induced *AccDnaJC28* mRNA expression in *A. cerana cerana* (**Figure 4C**), suggesting that *AccDnaJC28* participates in the UV-responsive chaperone network. This observation is consistent with the general notion that UV stress activates heat-shock/chaperone systems, as reported for *Hsp70* in human dermal fibroblasts (Roh et al., 2008), *Hsp27* in human corneal epithelial cells (Shi et al., 2006), *Hsp83*/*Hsp68*/*Hsp27* in *Tribolium castaneum* (Sang et al., 2012), and multiple *sHsps* in *Spodoptera frugiperda* (Yang et al., 2021). More relevantly, it resembles the UV-induced expression of *DnaJA1*, *DnaJB12*, *DnaJC3*, and *DnaJC8* in *A. cerana cerana* (Li et al., 2018; Zhang et al., 2019), indicating that *DnaJs* are collectively recruited during UV stress in this species.

In addition to the induction of *DnaJC28* expression by cold stress, heat stress and UV stress, we also discovered that the mRNA level of *DnaJC28* increased upon treatment with pesticides, including paraquat, beta-cypermethrin and abamectin (**Figures 4D–F**). Paraquat and beta-cypermethrin can harm honeybees even at low concentrations (Cousin et al., 2013; He et al., 2022; Phokasem et al., 2024). Abamectin is classified on commercial labels as highly toxic to honeybees (Bai and Ogbourne, 2016). The upregulated expression of *AccDnaJC28* may help *A. cerana cerana* withstand exposure to paraquat, beta-cypermethrin and abamectin and increase the likelihood of survival.

Multiple environmental stressors, such as cold, heat, and UV, as well as paraquat, beta-cypermethrin, and abamectin, can trigger increases in reactive oxygen species (ROS) levels in organisms (Finkel and Holbrook, 2000; Meng et al., 2009; Biova et al., 2025). ROS include hydroxyl radicals, superoxide anions and hydrogen peroxide (H_2_O_2_) (Nakai and Tsuruta, 2021). In all aerobic organisms, ROS are believed to be produced as part of normal oxidative metabolism. A certain level of ROS is necessary for host defense and cellular proliferation. However, high levels of ROS are detrimental, triggering oxidative stress and damage, damaging DNA, lipids, and proteins, and even accelerating age-related diseases and cell death (Finkel and Holbrook, 2000). In addition to the induction of its expression by exposures to cold treatment, heat treatment, paraquat, beta-cypermethrin, abamectin, and UV stress, we also found that the mRNA levels of *AccDnaJC28* increased upon H_2_O_2_ treatment (**Figure 4B**). Therefore, we suggest that the induction of a high level of *AccDnaJC28* expression is a potential protective strategy for coping with the increase in ROS levels induced by multiple environmental stresses and reducing oxidative damage. Furthermore, *AccDnaJC28* is a promising new biomarker for environmental stress, serving as an early indicator of exposure.

A sophisticated antioxidant defense system involving antioxidant genes and enzymes plays a crucial role in regulating overall ROS levels, counteracting ROS accumulation, and mitigating oxidative damage (Finkel and Holbrook, 2000; Somogyi et al., 2007). We found that knockdown of *AccDnaJC28* decreased the mRNA levels of antioxidant genes, such as *CYP336A1*, *GSTZ1*, and *TrxR1* (**Figures 7P–R**), and the enzymatic activities of antioxidant enzymes such as POD and T-SOD (**Figures 8A, B**). These results indicate that the ability of *AccDnaJC28* to decrease oxidative damage caused by multiple environmental stresses may be closely associated with the antioxidant defense system. Furthermore, the expression of many antioxidant genes was also upregulated when *AccDnaJC28* was knocked down (**Figures 7A–O**); this change may compensate for *AccDnaJC28* knockdown under oxidative stress to reduce damage to *A. cerana cerana*.

In conclusion, our results suggest that *AccDnaJC28* is a typical DnaJ protein that is located in the cytoplasm and may function as a novel biomarker of environmental stress. *AccDnaJC28* may reduce oxidative damage by activating the antioxidant defense system, thereby increasing *A. cerana cerana* survival under stress conditions. Our findings contribute to the understanding of honeybee defense mechanisms against diverse environmental stressors and provide a foundation for promoting honeybee health and well-being.

## Data Availability

The datasets presented in this study can be found in online repositories. The names of the repository/repositories and accession number(s) can be found in the article/**Supplementary Material**.

## Supplementary material

Supplementary Figure 1 Amino acid sequence alignment of AccDnaJC28 and other DnaJC28 proteins. Amino acids that are the same in all sequences are shown in black. The HPD motif is highlighted with red triangles. Multiple amino acid sequence alignment of AccDnaJC28 and other DnaJC28 proteins. AccDnaJC28: *A. cerana cerana* DnaJC28; AmDnaJC28: *A. mellifera* DnaJC28; BmDnaJC28: *Bombyx mori* DnaJC28; EcDnaJC28: *Equus caballus* DnaJC28; HsDnaJC28: *Homo sapiens* DnaJC28; MmDnaJC28: *Mus musculus* DnaJC28; XtDnaJC28: *Xenopus tropicalis* DnaJC28. The NCBI IDs of these proteins are listed in **Supplementary Table S2**.

Supplementary Table 1 Primers used in this study.

Supplementary Table 2 Protein information used in the evolutionary tree analysis and multiple amino acid sequence alignment in this study.
