# Supplementary material for: Functional analyses of Apis cerana cerana DnaJC28: a new biomarker for environmental stress
Source: Front Physiol. 2026 Oct 23;17:1945469. doi: 10.3389/fphys.2026.1945469 (PMC13739571; doi:10.3389/fphys.2026.1945469)
Supplement: Supplementary file 1 [file Table1.docx]

**Table S1. Primers used in this study.**

| **Primer sequence (5’–3’)** | **Description** |
| --- | --- |
| ATGACAATAAGATTTAATTCA | CDS primer of *AccDnaJC28*, forward |
| TTATTTATTAAAAATCGAAGAT | CDS primer of *AccDnaJC28*, reverse |
| AAGGAAAAAAGCGGCCGCATGACAATAAGATTTAATTCA | Primers used for constructing the pUAST-6×Myc-DnaJC28 eukaryotic expression vector, forward |
| GCTCTAGATTATTTATTAAAAATCGAAGATATC | Primers used for constructing the pUAST-6×Myc-DnaJC28 eukaryotic expression vector, reverse |
| GGATCCTAATACGACTCACTATAGGCTCTAGAAGTTGCAGAAGATTG | RNAi primer of *AccDnaJC28*, forward |
| GGATCCTAATACGACTCACTATAGGGTTTACCCATTCCTGGTAGATC | RNAi primer of *AccDnaJC28*, reverse |
| TAATACGACTCACTATAGGGCGAAGTGGAGAGGGTGAAGGTGA | RNAi primer of *GFP*, forward |
| TAATACGACTCACTATAGGGCGAGGTAAAAGGACAGGGCCATC | RNAi primer of *GFP*, reverse |
| ACAGCACCACAACATCGT | qRT–PCR primer of *AccDnaJC28*, forward |
| GATCCATGCCAAAACGTG | qRT–PCR primer of *AccDnaJC28*, reverse |
| TTATATGCCAACACTGTCCTTT | qRT–PCR primer of *β-actin*, forward |
| AGAATTGATCCACCAATCCA | qRT–PCR primer of *β-actin*, reverse |
| CGCAAAGAGAATGGGAAGG | qRT–PCR primer of *CYP4G11*, forward |
| CTTTTGTGTGACGGAGGTGC | qRT–PCR primer of *CYP4G1*, reverse |
| CGAACGCCGGTAGACCCTTG | qRT–PCR primer of *CDK5*, forward |
| CCTTGAGCGGGATGATAAAGTGG | qRT–PCR primer of *CDK5*, reverse |
| CAACACGCACAACCCGAC | qRT–PCR primer of *CDK5r*, forward |
| GGAAGTCTCTTAAACGGGTGC | qRT–PCR primer of *CDK5r*, reverse |
| GGTTTGAGAATTATACGCACTGC | qRT–PCR primer of *Trx1*, forward |
| GAGTAAGCATGCGACAAGGAT | qRT–PCR primer of *Trx1*, reverse |
| GGTTCGGTAGTACTTGTGGAC | qRT–PCR primer of *Trx2*, forward |
| GGACCACACCACATAGCAAAG | qRT–PCR primer of *Trx2*, reverse |
| GCGTTAACTGTTCGTGCTTT | qRT–PCR primer of *Tpx4*, forward |
| CAAGGAATCTATAACTCGCAAG | qRT–PCR primer of *Tpx4*, reverse |
| GGGGTATTCTATTTTCGCATCCA | qRT–PCR primer of *Tpx5*, forward |
| CCATTTACGATGAGAATCGACTGA | qRT–PCR primer of *Tpx5*, reverse |
| CGATGAGCACGGTTGGATTTCAC | qRT–PCR primer of *Hsp22.6*, forward |
| GGTTCTGCTGCTGTTTGGGTG | qRT–PCR primer of *Hsp22.6*, reverse |
| AAGTATTAGATCAGGGACGAG | qRT–PCR primer of *MsrB*, forward |
| TCTTCTTCTCTTCTCCTGCTGG | qRT–PCR primer of *MsrB*, reverse |
| CCAGAAGTAAAAGGACAAGTTCGT | qRT–PCR primer of *GSTO2*, forward |
| CCATTAACATCAACAAGTGCTGGT | qRT–PCR primer of *GSTO2*, reverse |
| CTTCTTAGTTATGGAGGTGTTG | qRT–PCR primer of *GSTS4*, forward |
| GCCATCTGAAATCGTAAAGAG | qRT–PCR primer of *GSTS4*, reverse |
| CGAAGGAGAAAACTATGTGGCAG | qRT–PCR primer of *GSTD*, forward |
| CGTAATCCACCACCTCTATCG | qRT–PCR primer of *GSTD*, reverse |
| GGACAACCAGTGACACAAGAA | qRT–PCR primer of *GSTT1*, forward |
| TGCTCCAATTCACATGCTCCA | qRT–PCR primer of *GSTT1*, reverse |
| AAACTATTCAACTTCAAGGACC | qRT–PCR primer of *SOD1*, forward |
| CACAAGCAAGACGAGCACC | qRT–PCR primer of *SOD1*, reverse |
| GCAAGGGATAAATTACGAATGTGTC | qRT–PCR primer of *MKK4*, forward |
| CTCCCTTTCATCCACTGTAGAAC | qRT–PCR primer of *MKK4*, reverse |
| TGTTCGGTTATTTGCCATTCC | qRT–PCR primer of *CYP336A1*, forward |
| GGTCTGCCAGTAAACTCTTCC | qRT–PCR primer of *CYP336A1*, reverse |
| CGAATCCGTCCATCAAAGCC | qRT–PCR primer of *GSTZ1*, forward |
| GTTGTGCCCATTCCTTTTTACG | qRT–PCR primer of *GSTZ1*, reverse |
| TCCTGCTATGTATGTCGC | qRT–PCR primer of *TrxR1*, forward |
| AGTTGCCATTTCCTGTTC | qRT–PCR primer of *TrxR1*, reverse |
| GCTGTAAATGAGGATTGTGAGCTG | qRT–PCR primer of *p38b*, forward |
| CGTCTCGTGGAGTACCCAGTAT | qRT–PCR primer of *p38b*, reverse |
| CCTGCACCTGAATTTTCCGG | qRT–PCR primer of *Tpx3*, forward |
| CTCGGTGTATTAGTCCATGC | qRT–PCR primer of *Tpx3*, reverse |

**Table S2. Protein information used in the evolutionary tree analysis and multiple amino acid sequence alignment in this study.**

| Protein name | Species origin | Protein ID in NCBI |
| --- | --- | --- |
| MmDnaJA2 | *Mus musculus* | NP_062768.1 |
| HsDnaJA2 | *Homo sapiens* | NP_005871.1 |
| PaDnaJA1 | *PaDnaJA1* | NP_001127102.1 |
| BtDnaJA1 | *Bos taurus* | NP_001015637.1 |
| DrDnaJA1 | *Danio rerio* | NP_955956.1 |
| XtDnaJA2 | *Xenopus tropicalis* | NP_001004807.1 |
| GgDnaJA2 | *Gallus gallus* | NP_001005841.1 |
| CvDnaJA2 | *Calliphora vicina* | XP_065357052.1 |
| BtDnaJA4 | *Bos taurus* | NP_001095590.2 |
| StDnaJA4 | *Salmo trutta* | XP_029607861.1 |
| LsDnaJA4 | *Lonchura striata* | XP_021403035.1 |
| DrDnaJB2 | *Danio rerio* | NP_001073462.1 |
| StDnaJB2 | *Scyliorhinus torazame* | XP_072325275.1 |
| VpDnaJB3 | *Vicugna pacos* | XP_031533649.2 |
| FaDnaJB6 | *Fopius arisanus* | XP_011303810.1 |
| VvDnaJB7 | *Vulpes vulpes* | XP_025873126.1 |
| MdDnaJB8 | *Myotis daubentonii* | XP_059560898.1 |
| SaDnaJB8 | *Strix aluco* | XP_074692679.1 |
| DdDnaJB8 | *Delphinus delphis* | XP_059877504.1 |
| EcDnaJC28 | *Equus caballus* | NP_001243864.2 |
| XtDnaJC28 | *Xenopus tropicalis* | NP_001015977.1 |
| BmDnaJC28 | *Bombyx mori* | NP_001296541.1 |
| AmDnaJC28 | *Apis mellifera* | XP_026295176.1 |
| DsDnaJC3 | *Drosophila suzukii* | XP_016939977.4 |
| DpDnaJC16 | *Danaus plexippus* | XP_032513324.1 |
| SfDnaJC17 | *Salarias fasciatus* | XP_029973140.1 |
| BcDnaJC19 | *Babesia caballi* | GIX60671.1 |
| PtDnaJC19 | *Pan troglodytes* | JAA02287.1 |
| CfDnaJC25 | *Cylas formicarius* | XP_060523802.1 |
| MmDnaJA3 | *Mus musculus* | NP_076135.3 |
| DaDnaJA3 | *Diadema antillarum* | XP_071483878.1 |
| NlDnaJA3 | *Nerophis lumbriciformis* | XP_061840495.1 |
| AbDnaJA1 | *Anopheles bellator* | XP_058054407.1 |
| LaDnaJB7 | *Loxodonta africana* | XP_023415666.1 |
| MlDnaJB8 | *Macrotis lagotis* | XP_074052166.1 |
| LkDnaJC18 | *Lepidochelys kempii* | XP_073212126.1 |
| SaDnaJC22 | *Sparus aurata* | XP_030278694.1 |
| XtDnaJC25 | *Xyrauchen texanus* | XP_051975089.1 |
| MmDnaJC27 | *Macaca mulatta* | NP_001244524.1 |
| MdDnaJC27 | *Mesoplodon densirostris* | XP_059962462.1 |
| TgDnaJC8 | *Taeniopygia guttata* | NP_001232243.1 |
| NiDnaJC5 | *Nymphalis io* | XP_050354544.1 |
| HsDnaJC28 | *Homo sapiens* | NP_001035282.1 |
| MmDnaJC28 | *Mus musculus* | NP_001093208.1 |
| XtDnaJC28 | *Xenopus tropicalis* | NP_001015977.1 |
